# Drug-drug interaction and acute kidney injury development: A correlation-based network analysis

**DOI:** 10.1371/journal.pone.0279928

**Published:** 2023-01-06

**Authors:** Wenjun Zhu, Erin F. Barreto, Jingshan Li, Hyo Kyung Lee, Kianoush Kashani

**Affiliations:** 1 Department of Industrial and Systems Engineering, University of Wisconsin-Madison, Madison, WI, United States of America; 2 Department of Pharmacy, Mayo Clinic, Rochester, MN, United States of America; 3 Department of Industrial Engineering, Tsinghua University, Beijing, China; 4 School of Industrial and Management Engineering, Korea University, Seoul, Republic of Korea; 5 Division of Nephrology and Hypertension, Department of Medicine, Mayo Clinic, Rochester, MN, United States of America; 6 Division of Pulmonary and Critical Care Medicine, Department of Medicine, Mayo Clinic, Rochester, MN, United States of America; Stanford University School of Medicine, UNITED STATES

## Abstract

**Background:**

Drug-induced nephrotoxicity is a relatively common preventable cause of acute kidney injury (AKI), providing early recognition and management. The pharmacokinetics or pharmacodynamics of drug-drug interactions may lead to additive or synergistic toxicity. The influx of new medications or off-label use of medications in the critical care setting can lead to additional nephrotoxicities, often challenging to predict or detect. This study evaluates the patterns of medication utilization, their combinations, and the related associations with AKI.

**Methods:**

We utilized correlation-based network analysis (CNA) to investigate the relationship between medications or their combinations with AKI in a large cohort of critically ill patients in a tertiary medical center between 2007 and 2018. Pairwise medication-AKI correlation analysis was performed to evaluate drug synergistic or additive effects. To investigate the inherent nephrotoxicity of medications, we further analyzed medications that were not paired with any other medications within 24 hours before or after their administration time (isolated medication analysis).

**Results:**

Among 147,289 ICU admissions, we identified 244 associations among 1,555 unique medication types. In pairwise analysis, 233 significant correlations were found among 13,150,198 medication pair instances. In isolated medication analysis, ten significant AKI associations were noted. When stratified by eGFR level, substantial differences between eGFR<90 vs. eGFR≥90 patients were observed. This highlights a need to determine eGFR as a risk factor for nephrotoxicity assessment when drug interactions are considered.

**Conclusions:**

This large-scale cohort study identified an artificial intelligence model to identify patient-agnostic relationships between medication or their pairs with AKI incidence among critically ill patients. It could be used as a continuous quality assurance tool to monitor drug-associated risk nephrotoxicity.

## Introduction

Acute kidney injury (AKI) is a relatively common complication of acute illness. The use of potentially nephrotoxic drugs is known to induce kidney dysfunction or injury, contributing to 14–21% of cases of AKI in critically ill patients [1–4]. Indeed, up to 22% of the 100 most administered drugs in adult intensive care are potentially nephrotoxic [5]. In addition, the number of new medications, including chemotherapeutics and biological drugs with potential nephrotoxicities, continues to increase exponentially [6,7]. Hence, healthcare providers need to be familiar with the drugs that may cause nephrotoxicity to select appropriate medications for their patient scenario and tailor the drug therapy program accordingly (e.g., discontinuation, dose adjustment). This essential information will facilitate preventive maneuvers, early identification, and directed disease management [8].

A standardized approach to defining drug-induced kidney disease (DIKD) has been proposed, but no pre-determined standardized nephrotoxin list exists. Moreover, the drug-drug interactions in DIKD are not well described [9]. For example, several drug combinations have been identified as potential concerns due to pharmacokinetic or pharmacodynamic drug interactions (e.g., vancomycin and piperacillin/tazobactam, aminoglycosides, and cephalothin, nonsteroidal anti-inflammatory drugs (NSAIDs) and radiocontrast, and cisplatin and aminoglycosides [10–13]). However, such associations are rarely detected in clinical trials and postmarketing analyses, while often, the primary focus of nephrotoxicity studies has been on single drug associations with AKI rather than combinations.

To understand multifaceted relationships between different drugs in the complex clinical environment in the intensive care unit (ICU), it is necessary to broadly evaluate drug utilization patterns in all patients and their association with AKI development in this setting [14,15]. Therefore, we aimed to understand the medication administration patterns, drug-drug interactions, and their impacts on AKI risk. We applied correlation-based network analysis (CNA) to investigate the medication administration patterns and their association with AKI risk by mining electronic medical record (EMR) data and stratifying it according to underlying factors such as age, sex, and estimated glomerular filtration rate (eGFR). This approach facilitates 1) identifying the medications most prescribed in ICUs among patients with AKI and 2) exploring the pattern of medication pairs most prescribed in ICUs among patients with AKI.

## Materials and methods

### Data description

The utilized data set consisted of medication administration records from patients admitted to the adult intensive care unit (ICU) of Mayo Clinic between January 2007 and May 2018. A total of 147,289 ICU admissions and 1,555 administered medication types were included in the study, which resulted in 16,603,882 medication administration instances.

### Data preprocessing

The serum creatinine (SCr) and urine output (UO) criteria proposed by the Kidney Disease: Improving Global Outcomes (KDIGO) were utilized to determine the AKI incidence and stages, described as 1) Stage 1: increase in SCr *≥* 1.5-2-fold from baseline or by *≥* 0.3 mg per dL, or UO of *≤* 0.5 mL per kg per hour for more than 6 hours, 2) Stage 2: increase in SCr *>*2-3-fold from baseline, or low UO of *≤* 0.5 mL per kg per hour for more than 12 hours, 3) Stage 3: increase in SCr *>3*-fold from baseline, or low UO of *≤* 0.5 mL per kg per hour for more than 24 hours.

The ICU admissions without available measured baseline SCr values were excluded from this study. All patients with evidence of AKI at the time of admission to the ICU were also excluded. Cases with AKI during the first 24 hours of ICU admission were also excluded to allow for sufficient time between drug exposure and AKI onset.

**Fig 1:**
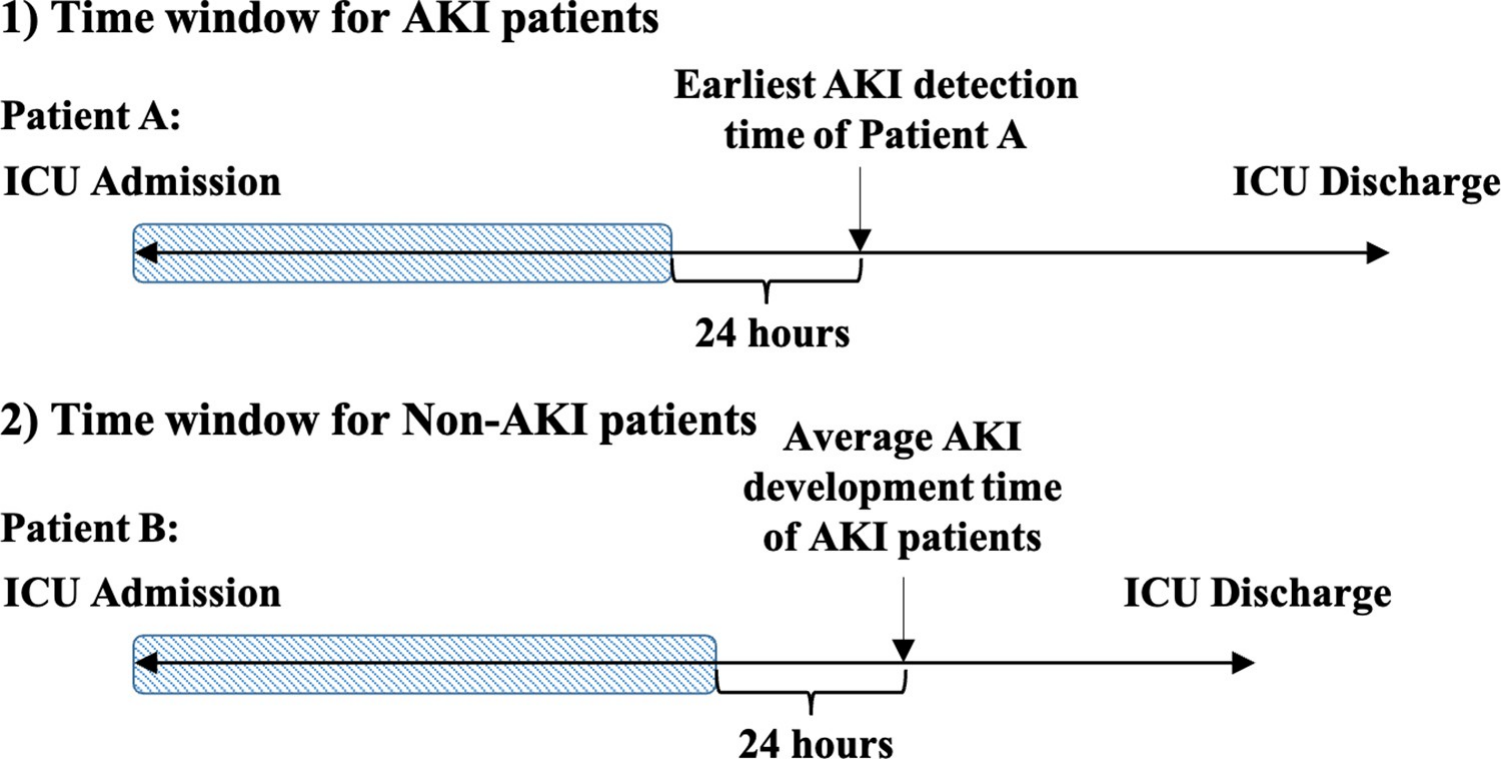
Time window for medication administration extraction. For the patient who develops AKI (Patient A), the time window ranges from ICU admission to 24 hours before that patient’s earliest AKI detection time. In contrast, for non-AKI patients (Patient B), the time window is from ICU admission to 24 hours before the average AKI development time for AKI patients.

Medication administration records and AKI stage-related variables for each ICU stay were listed and linked with a unique encounter ID in the original data set. Due to the numerous non-uniform dosage units and co-existence of continuous (e.g., fluid) and discrete (e.g., tablet) medication types, we only considered the counts of medication administered to the patient during their hospitalization at the ICU without considering dosages. The time of the first SCr or UO to meet AKI diagnosis was considered time zero for AKI patients. For patients who developed AKI, medications administered from ICU admission to 24 hours before the earliest AKI detection time were tagged as potentially correlated with AKI development (**Fig 1**).

For the non-AKI group of patients, we considered ICU admission time + average time of AKI development for the AKI group as time zero for exposure to medications with potential nephrotoxicity. Therefore, medication administrations from ICU admission time to 24 hours before the a-priori-defined time zero for AKI and non-AKI groups were extracted from the medical records (**Fig 1**).

Several medications were excluded from the analysis based on their administration route, including per rectum (PR), inhalation (IH), intra-ocular, topical, vaccinations, food products, and medications prescribed via gastrostomy tubes (GT; these mostly included multivitamins with minerals, lansoprazole, sodium phosphate, oxycodone, ferrous sulfate, ranitidine, atorvastatin, lisinopril, warfarin).

### Evaluation of medications associated with AKI

We used Pearson correlation coefficients to investigate the relationship between medications and AKI development. In addition, the Bonferroni correction was used to minimize Type I Errors.

**Fig 2:**
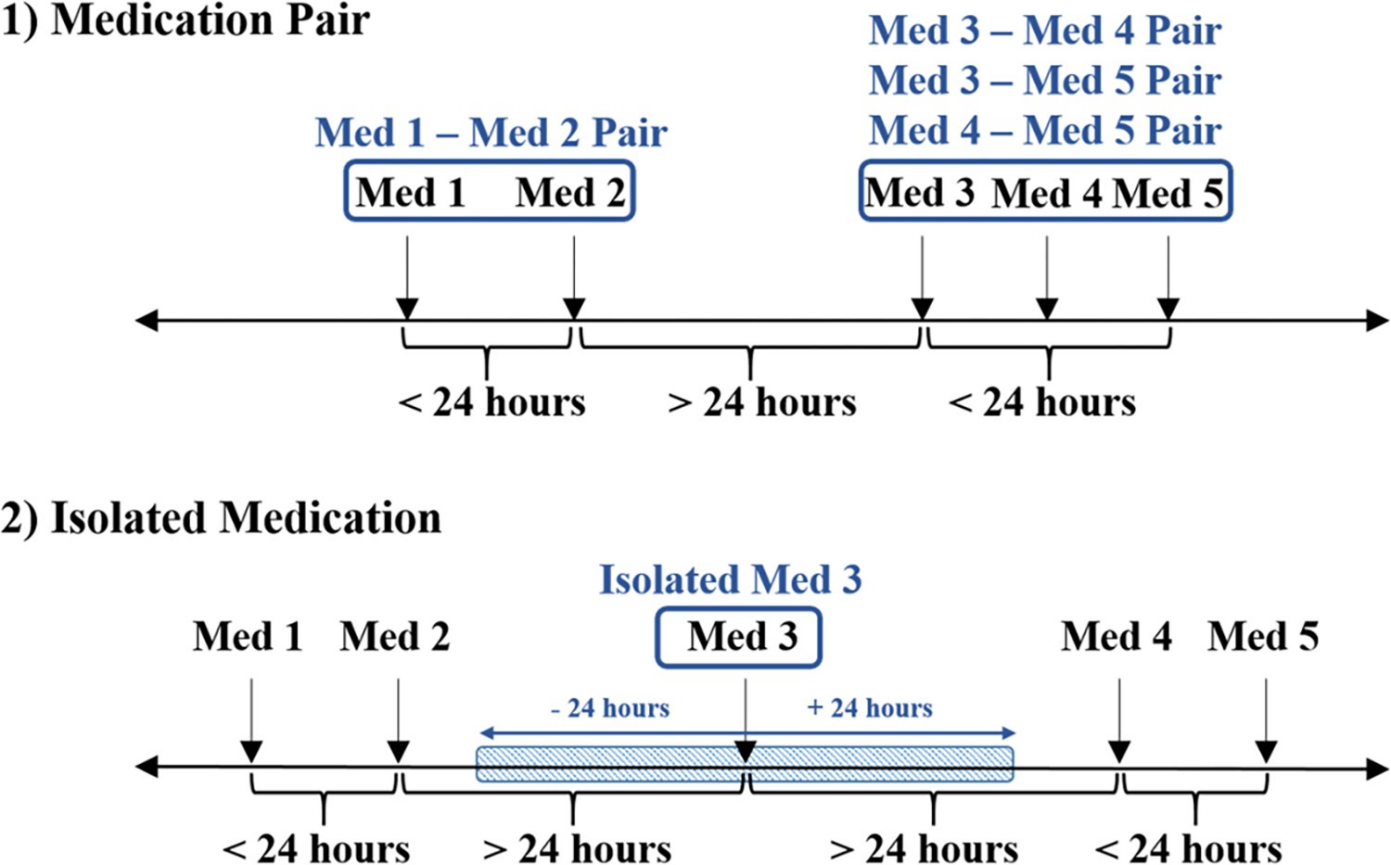
Medication pair and isolated medications. 1) Medication pairs are combinations of two medications administered during one ICU admission with a time interval of ≤24 hours. For example, medications 1 and 2 together and any combination of Medications 3, 4, and 5 are considered pairs. However, none of medications 1, 2 can form a pair with medications 3, 4, and 5, as they are not administered within 24 hours. 2) Isolated medications are not administered with any other medications in pairs, i.e., within 24 hours. For example, medication 3 is an isolated medication, as there are no other medications (medications 1, 2, 4, and 5) administered 24 hours before or after its administration.

**Fig 3:**
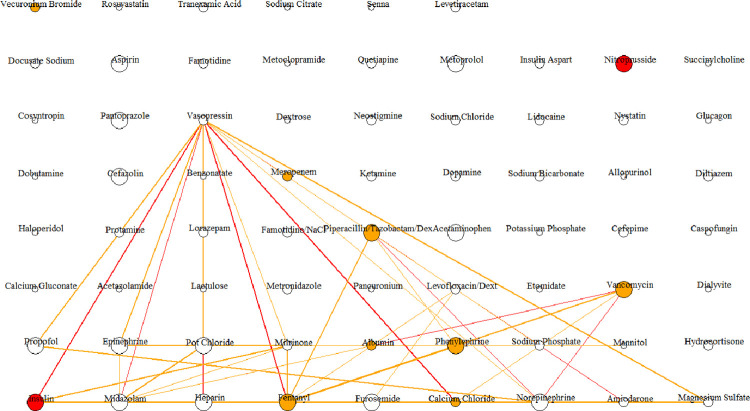
Hairball network diagram of medications pairs with a significant correlation with AKI. The 233 significant medication pairs, consisting of 69 unique medications, are visualized as a hairball network diagram where each vertex indicates a medication (69 unique medications resulting in 69 vertices). Each of the vertices is linked via an edge if two medications are medication pairs with a high AKI correlation (233 significant medication pairs resulting in 233 edges; however, the edges associated with Pearson similarity coefficients of <0.07 are eliminated for better visibility; for a full view of all edges, please see S2 Fig). The width of the edges represents the administration frequency (0 *−* 50; 50 *−* 500; *>*500). The color of the edges shows the strength of Pearson similarity coefficients for the medication pairs (correlation coefficients *>*0.08 is red, and 0.07*−*0.08 is orange). Stronger Pearson similarity coefficients indicate a more significant relationship between the prescription of isolated medications and AKI development. The size of the vertices represents the administration frequency of the single medications (0 *−* 20; 20 *−* 100; *>*100). The color of the vertices shows the strength of Pearson similarity coefficients for the isolated medications (correlation coefficient of *>*0.12 is red, 0.07*−* 0.12 is orange, and when there is no significant correlation, or medication was never administered alone, it is white).

We assessed medication-AKI correlation as 1) single medication-AKI correlation to identify the association between AKI and each administered medication, where we used Pearson similarity coefficients. The medications were then ranked based on the strength of their associations with AKI, and 2) pairwise medication-AKI correlation to evaluate the synergistic or additive effect of co-administered drugs. We defined the "medication pairs" as two medications administered during one ICU admission, with a time interval of *≤* 24 hours (**Fig 2)**, and 3) isolated medication-AKI correlation. While some medications have inherent nephrotoxicity, in others, the nephrotoxic effects are in the presence of another agent or medication. Therefore, "isolated medications" were those that could not be paired with any other medications within 24 hours before or after administration (**Fig 2**).

The correlation analyses were performed on the entire population and subgroups stratified by an estimated Glomerular filtration rate (eGFR) of below or above 90*mL/min/*1.73*m*^2^, calculated using the Modification of Diet in Renal Disease (MDRD) equation [16].

### Visualization of medication-AKI network

Due to the complexity of medication association with AKI, we utilized correlation-based network analysis (CNA) to investigate the medication administration patterns and their association with AKI. In CNA, a network can be represented as a graph of nodes connected in pairs by a rule through links. The R package "igraph" was utilized to draw a "hairball" network diagram and visualize the medication-AKI network. Each vertex indicates a medication, and two vertices would be linked together via an edge if two medications were within a "medication pair."

We used the size of the nodes and the width of the edges to represent the administration frequency of single or pairs of medications, respectively. In addition, the colors of the nodes and edges show the strength of correlation coefficients. The resulting weighted networks were analyzed using the tools of network theory for the entire population and the eGFR stratified subgroups.

We performed community detection via a fast greedy modularity optimization algorithm to identify clusters of medications that tend to be connected more densely when given in pairs. The strength of the correlation was taken into consideration as the attributed weight in finding the community structure. The communities were visualized in subgraphs within a global network characterized by higher connectivity between their components than in the other regions. Interpreting the complex medication network as a set of communities was used to identify clinically meaningful patterns that might have been hidden.

## Results

After excluding 47,887 ICU admissions and 16,269,007 medication administration instances, the final data set contained 99,402 ICU admissions (88,567 (89.10%) without AKI and 10,835 (10.90%) with AKI developed after 24 hours of ICU admission), 1,096 medication types and 334,875 medication administration instances administered before the AKI development or time zero for non-AKI patients (**S1 Fig**). The average AKI development time was 61 (±108) hours. Based on a-priori plans for non-AKI patients, the 61^st^ hour of ICU admission was selected as time zero.

### Correlation analysis

#### Single medication-AKI correlation analysis

**Table 1** lists the twenty most frequently administered medications associated with AKI. Two hundred forty-four medications were significantly correlated with AKI development. Among these drugs, eleven were included in the 20 most commonly administered medications, i.e., furosemide, heparin, vancomycin, potassium chloride, propofol, norepinephrine, insulin, piperacillin/tazobactam, fentanyl, midazolam, metoprolol.

**Table 1:** Top 20 frequently administered and significant AKI-correlated medications.

*Frequently prescribed*		*Associated with AKI*	
*Medication*	*Count (Frequency)*	*Medication*	*Pearson Coefficient*
Fentanyl	297861 (0.089)	Furosemide*	0.154323
Insulin	169056 (0.050)	Heparin*	0.148904
Propofol	147706 (0.044)	Vancomycin*	0.131278
Acetaminophen	133364 (0.040)	Potasium Chloride*	0.130062
Heparin	125716 (0.038)	Propofol*	0.125510
Nitroprusside	122993 (0.037)	Norepinephrine*	0.124628
Pot Chloride	96114 (0.029)	Albumin	0.110963
Phenylephrine	81824 (0.024)	Insulin*	0.104128
Oxycodone	81693 (0.024)	Piperacillin/Tazobactam Dextrose*	0.101780
Norepinephrine	81439 (0.024)	Calcium Chloride	0.100198
Metoprolol	81161 (0.024)	Nystatin	0.099618
Hydromorphone	71225 (0.021)	Cefepime	0.095724
Furosemide	56988 (0.017)	Vasopressin	0.095714
Epinephrine	53283 (0.016)	Fentanyl*	0.095669
Cefazolin	50895 (0.015)	Quetiapine	0.093315
Aspirin	41843 (0.012)	Midazolam*	0.093260
Piperacillin/Tazobactam Dextrose	41609 (0.012)	Levofloxacin/Dex	0.093146
Vancomycin	39225 (0.012)	Meropenem	0.088812
Pantoprazole	36894 (0.011)	Lidocaine	0.087702
Midazolam	35438 (0.011)	Metoprolol*	0.086261

#### Pairwise medication-AKI correlation analysis

The data set contained 13,150,198 medication pair instances, consisting of 124,198 unique medication pairs. The medication pairs were sorted based on their administration frequency (**Table 2)**. The top twenty medication pairs consisted of 12 unique medications, among which all except magnesium sulfate were included in the 20 most frequently prescribed single medication lists (**Table 1**).

**Table 2:** Top 20 frequently administered medication pairs.

*Medication Pairs*		*Count*	*Frequency*
*Medication #1*	*Medication #2*		
Acetaminophen	Fentanyl	50036	0.003805
Fentanyl	Propofol	41832	0.003181
Acetaminophen	Oxycodone	37846	0.002878
Fentanyl	Potassium Chloride	36127	0.002747
Acetaminophen	Heparin	35996	0.002737
Fentanyl	Heparin	34263	0.002606
Fentanyl	Oxycodone	32541	0.002475
Cefazolin	Fentanyl	31207	0.002373
Acetaminophen	Potassium Chloride	30725	0.002336
Fentanyl	Insulin	28800	0.002190
Acetaminophen	Aspirin	28257	0.002149
Acetaminophen	Metoprolol	28192	0.002144
Aspirin	Fentanyl	27426	0.002086
Fentanyl	Metoprolol	27086	0.002060
Aspirin	Heparin	26652	0.002027
Fentanyl	Furosemide	26377	0.002006
Furosemide	Pot Chloride	26368	0.002005
Fentanyl	Magnesium Sulfate	26341	0.002003
Heparin	Metoprolol	25043	0.001904
Aspirin	Furosemide	24784	0.001885

Two hundred thirty-three medication pairs were significantly correlated with the AKI development, consisting of 69 unique medications. Among those, 65 drugs were also included in the list of significant single medications. **Table 3** shows the top 20 drugs included in pairs and the list of single medications.

**Table 3:** Top 20 significant AKI-correlated medication pairs.

*Medication Pairs*		*Pearson Coefficient*
*Medication #1*	*Medication #2*	
Insulin	Midazolam	0.09678323
Midazolam	Vasopressin	0.09413781
Heparin	Norepinephrine	0.09215886
Insulin	Vasopressin	0.09058875
Fentanyl	Norepinephrine	0.08992984
Heparin	Vasopressin	0.08618485
Fentanyl	Vasopressin	0.08608049
Midazolam	Vancomycin	0.0846426
Furosemide	Midazolam	0.08395664
Calcium Chloride	Vasopressin	0.08361297
Midazolam	Norepinephrine	0.08287218
Norepinephrine	Piperacillin/Tazobactam Dextrose	0.08193319
Norepinephrine	Vancomycin	0.08110514
Amiodarone	Vasopressin	0.08060275
Magnesium Sulfate	Vasopressin	0.07895175
Propofol	Vasopressin	0.07812128
Midazolam	Milrinone	0.07636951
Norepinephrine	Vasopressin	0.07599747
Calcium Chloride	Insulin	0.07587109
Magnesium Sulfate	Midazolam	0.07581045

Interestingly, there was no overlap between Tables 2 and 3, which indicates that the most significant AKI-correlated medication pairs were not frequently prescribed in ICUs. Also, the strength of the correlation coefficients generally were weaker than those from the single medication analysis.

#### Isolated medication-AKI correlation analysis

Sixty-four of the 69 medications identified in the medication pair analysis were administered alone at least once (etomidate, benzonatate, cosyntropin, succinylcholine, and senna were only applied in pairs), resulting in 2,752 medication administration instances. Among the 64 medications, ten were significantly correlated with AKI development (Bonferroni corrected α = 0.01/64 = 1.56E – 04; **Table 4**). All ten medications were also significantly correlated with AKI during the single medication-AKI correlation analysis. Except for nitroprusside, all drugs were included in the top 20 AKI-correlated drugs indicated in **Table 1**. Among those nine drugs, five medications (insulin, fentanyl, vancomycin, phenylephrine, and piperacillin/tazobactam/dex) were among the 20 most frequently prescribed medications.

**Table 4:** Significant AKI-correlated isolated medications.

*Isolated Medication*	*Pearson Coefficient*
Insulin^*,**^	0.142192
Nitroprusside	0.123275
Albumin*	0.118331
Fentanyl^*,**^	0.110381
Vancomycin^*,**^	0.092251
Phenylephrine^*,**^	0.091323
Vecuronium Bromide*	0.077508
Piperacillin/Tazobactam Dextrose^*,**^	0.077496
Calcium Chloride*	0.077086
Meropenem*	0.077086

Among the 233 significant medication pairs, in 16 pairs, both medications in the pairs were significantly correlated with AKI in isolated analysis. In 86 pairs, only one medication in the pairs was identified as significant isolated medication, and finally, in 131 pairs, none of the medications were among the significant isolated medications. More than half of the significant medication pairs did not contain any significant isolated drugs. Also, 118 among these 131 pairs did not include any of the five medications which have never been administered alone. Hence, in these 118 medication pairs, individual drugs were not correlated with AKI when given independently but significantly associated with AKI when given in pairs, suggesting potential nephrotoxic drug-drug interactions (**Table 5;** the top 11 could also be found in **Table 3**).

**Table 5:** Top 20 significant AKI-correlated medication pairs that do not consist of any significant isolated medications.

*Medication Pair* *Medication #1*	*Medication Pair* *Medication #2*	*Pearson Coefficient*
Midazolam	Vasopressin	0.09413781
Heparin	Norepinephrine	0.09215886
Heparin	Vasopressin	0.08618485
Furosemide	Midazolam	0.08395664
Midazolam	Norepinephrine	0.08287218
Amiodarone	Vasopressin	0.08060275
Magnesium Sulfate	Vasopressin	0.07895175
Propofol	Vasopressin	0.07812128
Midazolam	Milrinone	0.07636951
Norepinephrine	Vasopressin	0.07599747
Magnesium Sulfate	Midazolam	0.07581045
Epinephrine	Milrinone	0.075703
Epinephrine	Vasopressin	0.07552481
Potassium Chloride	Vasopressin	0.07518455
Heparin	Midazolam	0.07503072
Magnesium Sulfate	Norepinephrine	0.07487292
Milrinone	Vasopressin	0.07457858
Midazolam	Potassium Chloride	0.07357606
Furosemide	Levofloxacin Dextrose	0.07235405
Milrinone	Potassium Chloride	0.0714607

#### Visualization of medication-AKI network

To better visualize the impact of medication pairs and isolated medications on AKI development, we drew the "hairball" network diagram to visualize the 233 pairs, as shown in **S2 Fig**. **Fig 3** only shows the colored edges to visualize the strength of correlated medication pairs.

**Fig 4:**
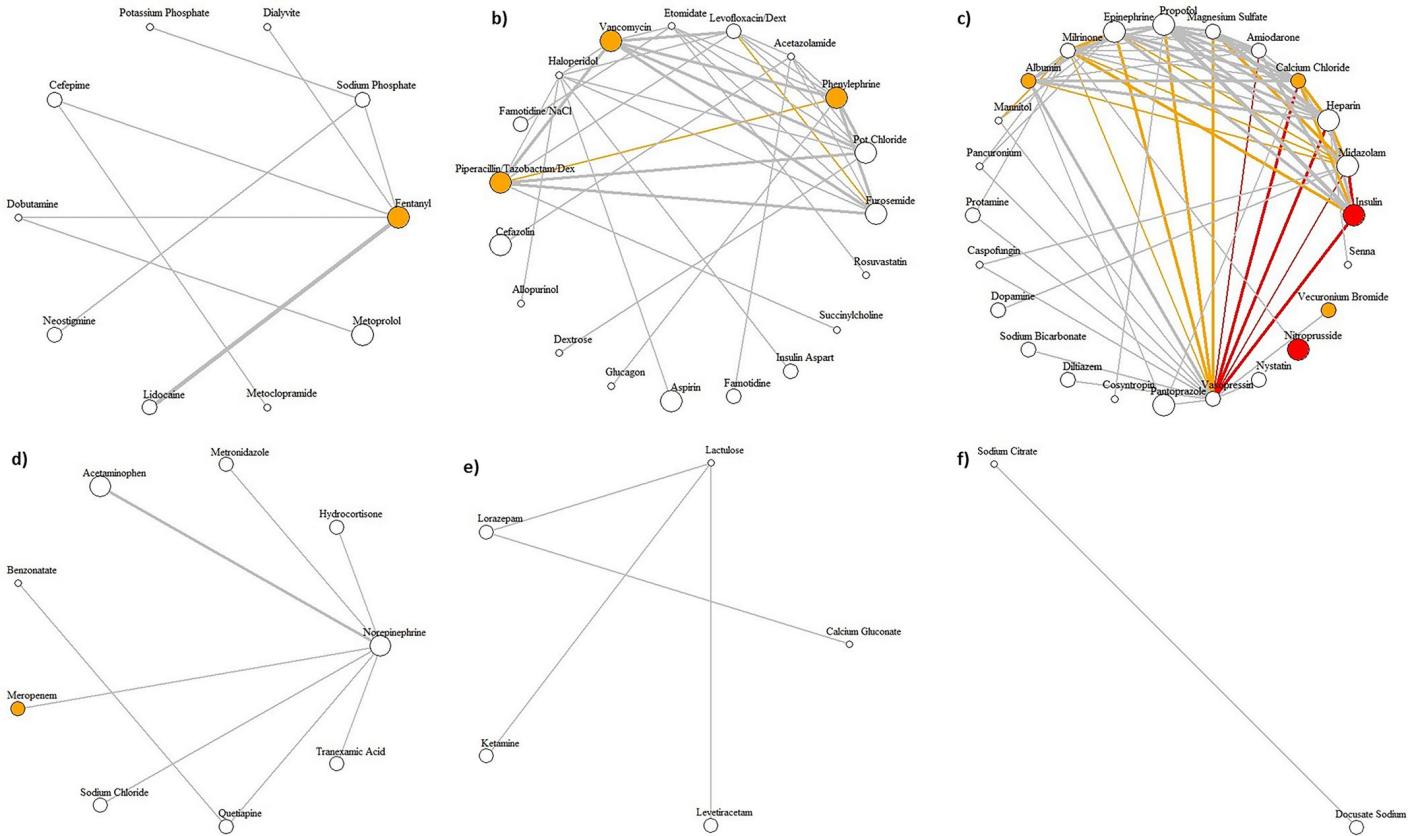
Hairball plots for medication clusters. We identified six communities of medication clusters (from a to f), where each cluster is visualized as a hairball network diagram. The width of the edges represents the administration frequency (0 *−* 50; 50 *−* 500; *>*500). The color of the edges shows the strength of Pearson similarity coefficients for the medication pairs (correlation coefficients *>*0.08 is red, and 0.07*−*0.08 is orange). Stronger Pearson similarity coefficients indicate a more significant relationship between the prescription of isolated medications and AKI development. The size of the vertices represents the administration frequency of the single medications (0 *−* 20; 20 *−* 100; *>*100). The color of the vertices shows the strength of Pearson similarity coefficients for the isolated medications (correlation coefficient of *>*0.12 is red, 0.07*−* 0.12 is orange, and when there is no significant correlation, or medication was never administered alone, it is white).

We identified six medication clusters to explore their relationship with AKI (**Fig 4**). We identified several patterns in our analytical approach, including 1) unlike all the AKI-correlated drugs identified in the isolated medication analysis that were among the most frequently prescribed drugs, e.g., insulin and nitroprusside, not all the medication pairs were among the frequently prescribed drugs, e.g., sodium phosphate/vasopressin. In addition, frequently prescribed medications or medication pairs were not necessarily correlated with AKI; 2) indeed, in medication pair analysis, some drugs were not associated with AKI when prescribed alone, e.g., vasopressin and sodium phosphate. In this case, potential nephrotoxicity due to drug-drug interactions could be present; 3) cluster 1 (**Fig 4A**) medications are associated with AKI both in pair and isolated analyses. Therefore, they could have additive or synergistic effects on AKI development. **Fig 4A** shows that most medications strongly correlated with AKI were also frequently prescribed. This notion provides a path to highlight practice patterns that could be evaluated to mitigate the nephrotoxicity burden; 4) **Fig 4B** shows medication pairs that are commonly prescribed and have less association with AKI; 5) The rest of the clusters (**Fig 4C–4F**) only contained no or one significant isolated medication without correlation with AKI. These clusters included single or paired medications that were not commonly prescribed; 6) **Table 6** lists the drugs highlighted in **Fig 3** with connections with at least ten other medications, indicating pairs associated with AKI. For example, while vasopressin contributes to most medication pairs, it is not included in the list of single medications related to AKI development. This finding may indicate that its utilization and relationship with AKI should likely be viewed as synergistic or additive rather than a nephrotoxin itself. Other medications that followed the same patterns included midazolam, heparin, propofol, milrinone, potassium chloride, amiodarone, and epinephrine. On the other hand, medications like fentanyl, calcium chloride, phenylephrine, vancomycin, piperacillin/tazobactam dextrose, albumin, and insulin were frequently prescribed, contributed to more than ten pairs, and as single medications were also associated with AKI; 7) **Fig 3** shows drugs with strong AKI correlation (ten medications were included in 43 medication pairs associated with AKI). Some of these medications contributed in limited pairs, e.g., nitroprusside and vecuronium bromide in one pair and meropenem in four pairs. These classes of medications likely are nephrotoxic as a single medication rather than a contributor to AKI in pairs. Within 43 identified medication pairs strongly associated with AKI, both medications were associated with AKI in 6 pairs, only one medication was associated with AKI in 13 pairs, and none of the medications were nephrotoxic in isolation in 24 pairs. Among the 14 pairs with the strongest AKI correlations, six pairs did not have any medications associated with AKI in isolation, i.e., midazolam-vasopressin, heparin-norepinephrine, heparin-vasopressin, furosemide-midazolam, midazolam- norepinephrine, amiodarone-vasopressin.

**Table 6:** Medications with high node degrees.

*Node (Medication)*	*Degree*	*Node (Medication)*	*Degree*	*Node (Medication)*	*Degree*
Vasopressin*	33	Phenylephrine^**^	18	Levofloxacin Dextrose	13
Norepinephrine	28	Potassium Chloride*	17	Insulin^**^	12
Fentanyl^**^	24	Vancomycin^**^	17	Etomidate	12
Midazolam*	23	Amiodarone*	15	Furosemide	11
Heparin*	18	Epinephrine*	15	Haloperidol	11
Calcium Chloride^**^	18	Piperacillin/Tazobactam Dextrose^**^	14	Lorazepam	10
Propofol*	18	Magnesium Sulfate	13		
Milrinone*	18	Albumin	13		

* Medications that contribute to the medication pairs associated with AKI but are not included on the list of nephrotoxins when used alone.

** Medication frequently prescribed, contributed in more than ten pairs of medications associated with AKI, and as single medications were also associated with AKI.

### Subgroup analysis

**Table 7** shows the results of subgroup analysis after stratification based on eGFR of 90 ml/min/1.73 m^2^ (Chronic kidney disease [CKD] with eGFR <90 ml/min/1.73 m^2^ vs. non-CKD with eGFR≥90 ml/min/1.73 m^2^).

**Table 7:** Number of medication instances in GFR subgroups.

	*non-AKI* *N*	*AKI* *N*
***eGFR <90***	1,225,594	279,969
***eGFR ≥* 90**	1,525,654	317,536

Abbreviations: eGFR, estimated glomerular filtration rate.

#### Single medication-AKI correlation analysis

Nine hundred eighty-seven unique medications were prescribed among CKD patients, with 186 (19%) significantly correlated with AKI. Among 988 unique medications, 191 (19%) were significantly associated with AKI in patients without CKD. **Table 8** lists the top 20 medications of each subgroup associated with AKI. Sixteen medications were common in both subgroups. While there were a lot of similarities between the AKI-associated medications in the subgroups, midazolam, meropenem, and metoprolol were more prevalent in the subset with CKD vs. lidocaine among non-CKD patients.

**Table 8:** Top 20 significant AKI-correlated medications for two subgroups.

*GFR<90 Group*		*GFR≥* 90 *Group*	
*Medication*	*Pearson Coefficient*	*Medication*	*Pearson Coefficient*
Furosemide*	0.156417	Furosemide*	0.157797
Heparin*	0.151747	Heparin*	0.14804
Vancomycin*	0.144074	Potassium Chloride*	0.132135
Norepinephrine*	0.136063	Propofol*	0.12224
Potassium Chloride*	0.130368	Vancomycin*	0.122034
Propofol*	0.130135	Norepinephrine*	0.116064
Insulin*	0.122824	Albumin*	0.112499
Calcium Chloride*	0.114971	Nystatin*	0.105866
Albumin*	0.110996	Piperacillin/Tazobactam Dextrose*	0.100413
Vasopressin*	0.110125	Quetiapine*	0.091413
Midazolam^**^	0.105243	Fentanyl*	0.091391
Piperacillin/Tazobactam Dextrose*	0.103455	Cefepime*	0.090986
Cefepime*	0.101895	Lidocaine^***^	0.089679
Fentanyl*	0.100948	Levofloxacin/Dext*	0.089573
Nystatin*	0.100707	Insulin*	0.088559
Meropenem**	0.097692	Calcium Chloride*	0.08819
Levofloxacin Dextrose*	0.097596	Sodium Chloride	0.086553
Quetiapine*	0.0957	Milrinone	0.086109
Epinephrine	0.092968	Vasopressin*	0.084579
Metoprolol^**^	0.091587	Metronidazole	0.084005

* Meications prescribed in both subgroups.

** Medications mostly used among those with chronic kidney disease with GFR*<*90 ml/min/1.73 m^2^.

*** Medications mostly used among those with chronic kidney disease with GFR*≥*90 ml/min/1.73 m^2^.

#### Pairwise medication-AKI correlation analysis

In the CKD subgroup, 42,661 unique medication pairs were used, with 205 pairs, including 71 unique medications, being significantly correlated with AKI. The non-CKD subgroup received 26,806 unique medication pairs, with 36 pairs (30 unique medications) significantly correlated with AKI. **Table 9** lists the top 20 AKI-associated medication pairs in each subgroup. The two subgroups did not share any of the top 20 AKI-associated medication pairs. Ten AKI-associated medication pairs among CKD patients were also found in the top 20 AKI-associated medication pairs in all patients. **Table 8** lists the top 20 medications of each subgroup that were associated with AKI.

**Table 9:** Top 20 significant AKI-correlated medication pairs for two subgroups.

*CKD*, *GFR<90 Group*			*Non-CKD GFR≥* 90 *Group*		
*Medication Pairs*		*Pearson Coefficient*	*Medication Pairs*		*Pearson Coefficient*
*Medication 1*	*Medication 2*		*Medication 1*	*Medication 2*	
Acetazolamide	Fentanyl	0.1106708	Etomidate	Vancomycin	0.13013489
Insulin*	Vasopressin*	0.1042989	Furosemide	Levofloxacin/Dext	0.12517537
Calcium Chloride*	Vasopressin*	0.10319643	Heparin	Senna	0.12332817
Heparin*	Norepinephrine*	0.10296665	Meropenem	Potassium Chloride	0.12137292
Milrinone	Vasopressin	0.09993951	Furosemide**	Midazolam**	0.12083589
Midazolam*	Vasopressin*	0.09904047	Metronidazole	Vasopressin	0.11958312
Fentanyl*	Vasopressin*	0.0989875	Sodium Chloride	Vasopressin	0.11876071
Fentanyl*	Norepinephrine*	0.09895096	Fentanyl	Levofloxacin/Dext	0.11670254
Epinephrine	Vasopressin	0.0981826	Dopamine	Pantoprazole	0.11633499
Propofol*	Vasopressin*	0.09743555	Furosemide	Vancomycin	0.11594301
Acetazolamide	Potassium Chloride	0.09513354	Cefepime	Vasopressin	0.1143419
Insulin	Milrinone	0.0942025	Etomidate	Piperacillin/Tazobactam Dextrose	0.11387659
Amiodarone*	Vasopressin*	0.09379757	Etomidate	Pot Chloride	0.11345592
Calcium Chloride	Milrinone	0.09313112	Midazolam**	Vancomycin**	0.11332864
Magnesium Sulfate*	Vasopressin*	0.09185513	Etomidate	Furosemide	0.11209237
Epinephrine	Milrinone	0.09170968	Etomidate	Phenylephrine	0.11147296
Insulin*	Midazolam*	0.09148049	Etomidate	Vasopressin	0.11103668
Acetazolamide	Lorazepam	0.08859342	Vancomycin	Vasopressin	0.11098672
Potassium Chloride	Vasopressin	0.08796814	Norepinephrine**	Piperacillin/Tazobactam Dextrose**	0.11006394
Acetazolamide	Furosemide	0.08789809	Norepinephrine	Sodium Chloride	0.10801834

* AKI-associated medication pairs that were common between the CKD subgroup and the whole cohort.

** ***AKI***-associated medication pairs that were common between the non-CKD subgroup and the whole cohort.

#### Isolated medication-AKI correlation analysis

In the CKD cohort, 58 of the 71 unique medications were administered alone at ***least*** once, and six were ***significantly*** correlated with AKI. In the non-CKD subgroup, all unique medications were administered alone (N = 30), and 6 ***of*** them were significantly associated with AKI (**Table 10**). Insulin was the only medication that was common among both subgroups.

**Table 10:** Significant AKI-correlated isolated medications for two subgroups.

*eGFR<90 Group*		*eGFR≥* 90 *Group*	
*Medication*	*Pearson Coefficient*	*Medication*	*Pearson Coefficient*
Vancomycin	0.132819	Insulin	0.113852
Nitroprusside	0.129079	Calcium Chloride	0.110098
Fentanyl	0.122475	Piperacillin/Tazobactam Dextrose	0.08078
Insulin	0.121948	Epinephrine	0.065386
Albumin	0.115707	Meropenem	0.065079
Phenylephrine	0.11045	Norepinephrine	0.065079

Abbreviations: eGFR, estimated glomerular filtration rate.

## Discussion

This large-scale retrospective analysis presents methods for identifying medications and drug combinations associated with AKI using correlation-based network analysis. We found 244 out of 1,096 drugs are possibly correlated with the AKI development, among which 10 of them are found to be significantly correlated when administered alone (i.e., significant isolated medications).

Our results should not be interpreted as a causal relationship between the identified drugs with AKI. Obviously, several identified drugs are used among patients with a high risk of AKI, e.g., sedatives and opioids used for mechanically ventilated patients. Instead, this study aimed to develop a model that periodically assesses practice patterns and identifies correlations between medications and medication pairs with AKI in each institution. This is particularly needed to identify potential nephrotoxins or nephrotoxic pairs amid changing practices and a deluge of new medication arrivals. If this model can identify potential strong correlations with AKI and these associations could be validated in separate studies, clinicians would have the chance to conduct quality improvement projects to change practice patterns and choose potentially less nephrotoxic drugs.

Among the ten significant isolated medications, vancomycin and piperacillin/tazobactam are known nephrotoxic drugs derived from the Nephrotoxic Injury Negated by Just-in-Time Action (NINJA) collaborative [17]. We also conducted a literature search to find relevant research papers that investigated the relationships between medications and AKI using the following keywords "AKI," "acute kidney injury," and "acute renal failure." Through the literature search, we found studies that investigated the associations between nitroprusside, phenylephrine, meropenem, and AKI [18–20], which matched our findings. However, as our goal was to identify potential associations that are not yet understood, future studies should focus on highlighted drugs, including insulin, albumin, fentanyl, vecuronium bromide, and calcium chloride, for their potential nephrotoxicities. These medications could be suitable candidates for clinical drug-safety studies to investigate possible associations or causal relationships with AKI, either directly or indirectly.

To verify the results of the medication pair analysis, we compared our findings with known drug interactions from DrugBank, which includes drug interactions with negative effects, by considering product labels and evidence in the literature [21]. Among the top 20 significant medication pairs that did not contain any significance in isolated medication analysis (**Table 5**), nine pairs (i.e., furosemide-midazolam, milrinone-midazolam, potassium chloride-midazolam, furosemide-levofloxacin, and milrinone-potassium chloride pairs are known to induce drug interactions that could affect serum level) were suggested to have drug-drug interactions by Drug Bank. Further studies are needed to investigate the drug-drug interactions on the medication pairs we identified as correlated with AKI but not reported in the literature (i.e., midazolam-vasopressin, heparin-norepinephrine, midazolam-norepinephrine, amiodarone-vasopressin).

We also incorporated basic patient characteristics, including age, sex, and eGFR, to stratify AKI risk categories and conduct subgroup analyses. In subgroup analyses, the unique single medications associated with AKI were similar between patients with and without an eGFR < 90 mL/min/1.73m^2^, but there were substantial differences when medication pairs were compared (4,353,466 for eGFR*<*90 vs. 3,234,548 for eGFR*≥* 90). This finding could indicate the presence of kidney dysfunction as an effect modifier in drug-drug interactions. This notion was even more palpable when we assessed the isolated medications. These data highlight a need to determine eGFR when drug interactions are considered a risk factor for nephrotoxicity assessment.

In a systematic review, the authors reported poor quality of available evidence of drug class combinations and their association with AKI development due to a lack of well-designed studies. Meanwhile, they demonstrated the literature regarding the impact of a combination of nonsteroidal anti-inflammatory drugs and diuretics with or without additional renin-angiotensin-aldosterone agents on AKI to have an overall higher quality of evidence [22]. Our study, however, showed differing results, likely due to multiple factors. These factors could include the lack of use of a unified AKI definition and focus on the effects of combining nephrotoxic drug classes instead of all possible administered drugs.

Nishata et al. used the association rules method to identify drug combinations associated with AKI in a large cohort of patients in New Zealand [23]. They reported several medication classes, including antimicrobials, nonsteroidal anti-inflammatory drugs, and opioids linked to AKI incidence. Unlike our study, the authors evaluated drug classes instead of specific medications. Ahmed and colleagues conducted a retrospective cross-sectional study in the pediatric ICU to assess potential associations between nephrotoxic drugs and the risk of developing AKI. They reported vancomycin as the most common single nephrotoxin, a combination of vancomycin and colistin as dual nephrotoxic agents, and vancomycin, colistin, and amphotericin B as a triple nephrotoxic combination [24]. The authors analyzed the drugs with known nephrotoxic activities rather than all possible administered drugs. The authors used the serum creatinine criterion to define AKI in a study to assess the association between nephrotoxic drug combinations and AKI for infants in ICU. They reported a combination of gentamicin and indomethacin to be more nephrotoxic when compared with furosemide and tobramycin or vancomycin and piperacillin-tazobactam [25]. However, the authors only evaluated a pre-determined list of drug combinations with known nephrotoxicity characteristics similar to the other investigations. In addition, the study time window was limited to the duration of the exposure to the studied combination therapy.

The main strength of our study is its exhaustive inclusion of all medications administered to a large cohort of patients regardless of their previously known nephrotoxicity characteristics. This allowed us to screen all medications used in 147,289 ICU admissions to discover any potential relationship with AKI. In addition, among the medications with a high association with AKI in single-drug analyses, we investigated their inherent nephrotoxicity by evaluating the effect of drug interactions through the "isolated medication analysis."

This study, similar to any retrospective study, has several limitations. First, we cannot infer any causal relationship. Second, as this analysis was conducted in a single center, its generalizability may be limited. Third, the study could not account for the dose of drug administered, potentially impacting our findings. ’Type A’ toxicities (dose-dependent) may have a different pattern than ’type B’ toxicities (idiosyncratic). Lastly, as we tried to limit individual patient characteristics entering our analyses, we could not assess the underlying reasons for the medication administrations, which could independently affect the risk of AKI. We need to emphasize that the primary purpose of this study is not to identify nephrotoxins for individual patients, but we aimed to develop a tool to assess the practice patterns related to AKI incidence. Such a tool or dashboard could facilitate a data-driven identification of candidate nephrotoxins suitable for future studies or scrutiny.

## Conclusions

This study developed several models to identify prescription patterns of unique or medication pairs clustered around AKI episodes. Our results may provide a platform to point to practice patterns that need to be changed or a novel understanding of the nephrotoxicity of medications or pairs of drugs that need further evaluation. We also found substantial differences in the clusters when CKD patients were compared with non-CKD patients. The utility of this tool in advancing the quality of care among hospitalized patients or research projects should be assessed in the future.

## Supporting information

S1 Fig Patient recruitment flow chart. (TIF)

S2 Fig Hairball network diagram for 233 significant medication pairs. The 233 significant medication pairs, consisting of 69 unique medications, are visualized as a hairball network diagram where each vertex indicates a medication (69 unique medications resulting in 69 vertices). Each of the vertices is linked via an edge if two medications are medication pairs with a high AKI correlation (233 significant medication pairs resulting in 233 edges). The width of the edges represents the administration frequency (0 *−* 50; 50 *−* 500; *>*500). The color of the edges shows the strength of Pearson similarity coefficients for the medication pairs (correlation coefficient of *>*0.08 is red, 0.07*−*0.08 is orange, and *<*0.07is grey). The size of the vertices represents the administration frequency of the single medications (0 *−* 20; 20 *−* 100; *>*100). The color of the vertices shows the strength of Pearson similarity coefficients for the isolated medications (red: Correlation coefficients *>*0.12; orange: 0.07*−* 0.12; white: Not significant or never administered alone).
(TIF)

S1 File (CSV)
