# Supplementary figures and images for: Drug-drug interaction and acute kidney injury development: A correlation-based network analysis
Source: PLoS One. 2023 Jan 6;18(1):e0279928. doi: 10.1371/journal.pone.0279928 (PMC9821414; doi:10.1371/journal.pone.0279928)

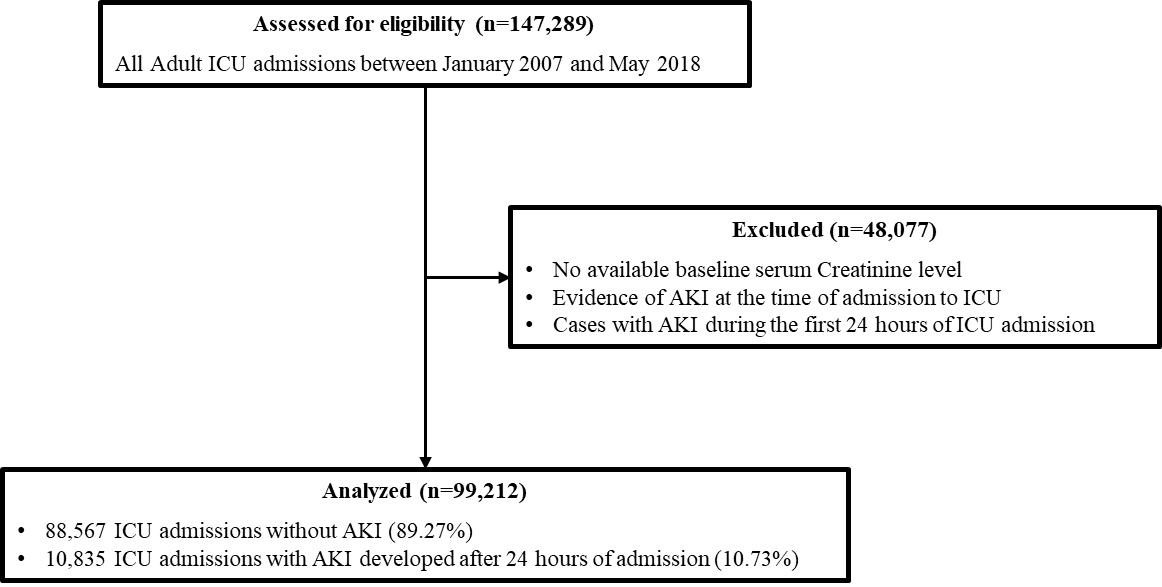

Supplement: S1 Fig — (TIF) [file pone.0279928.s001.tif]

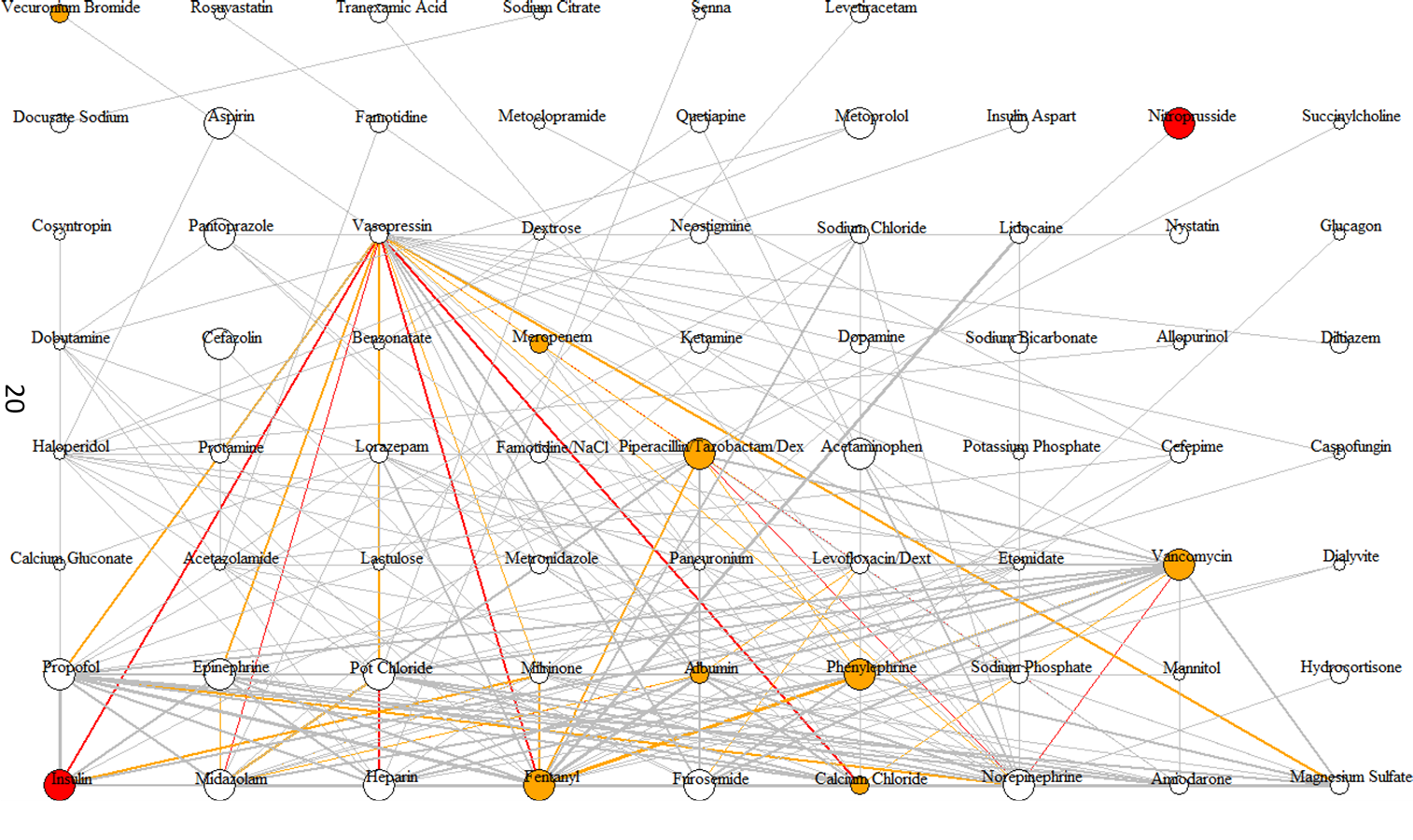

Supplement: S2 Fig — The 233 significant medication pairs, consisting of 69 unique medications, are visualized as a hairball network diagram where each vertex indicates a medication (69 unique medications resulting in 69 vertices). Each of the vertices is linked via an edge if two medications are medication pairs with a high AKI correlation (233 significant medication pairs resulting in 233 edges). The width of the edges represents the administration frequency (0 − 50; 50 − 500; >500). The color of the edges shows the strength of Pearson similarity coefficients for the medication pairs (correlation coefficient of >0.08 is red, 0.07−0.08 is orange, and <0.07is grey). The size of the vertices represents the administration frequency of the single medications (0 − 20; 20 − 100; >100). The color of the vertices shows the strength of Pearson similarity coefficients for the isolated medications (red: Correlation coefficients >0.12; orange: 0.07− 0.12; white: Not significant or never administered alone). (TIF) [file pone.0279928.s002.tif]
